# Whole-Genome Re-Sequencing of *Corylus heterophylla* Blank-Nut Mutants Reveals Sequence Variations in Genes Associated With Embryo Abortion

**DOI:** 10.3389/fpls.2019.01465

**Published:** 2019-11-13

**Authors:** Yunqing Cheng, Siqi Jiang, Xingzheng Zhang, Hongli He, Jianfeng Liu

**Affiliations:** Jilin Provincial Key Laboratory of Plant Resource Science and Green Production, Jilin Normal University, Siping, China

**Keywords:** whole-genome re-sequencing, *Corylus heterophylla*, blank-nut mutants, embryo abortion, single-nucleotide polymorphism

## Abstract

Yield loss in the economically important hazelnut (*Corylus* spp.) occurs through the frequent formation of blank nuts. Although the condition is associated with embryo abortion, we have not yet identified the regulatory genes involved. Therefore, this study aimed to determine the genes related to embryo abortion in hazel. We performed whole-genome re-sequencing and single-nucleotide polymorphism (SNP) analysis on four mutant hazelnut trees (Empty1 to Empty4, *C. heterophylla*) bearing blank nuts and four wild-type trees (Full1 to Full4, *C. heterophylla*). A paired comparison of Empty1 vs. Full1, Empty2 vs. Full2, Empty3 vs. Full3, and Empty4 vs. Full4, along with the intersection of Empty1 to Empty4, revealed 3 081 common SNPs in the four blank-nut mutants. Of these, 215 synonymous SNPs in exonic regions were distributed across 178 candidate genes. Heterozygosity analysis showed that average homozygous and heterozygous SNP ratios were respectively 0.409 and 0.591 in the samples. According to Gene Ontology classification, candidate genes were enriched in the categories of binding, catalysis, molecular transducer, transporter, and molecular function regulator. Among these, 18 of 178 genes had homozygous SNPs in Empty1–4. Cis elements in the promoter region of starch synthase 4 (SS4) contain the RY-element, implying seed-specific expression. Starch granules were absent from Empty1–4 cotyledon cells, but abundantly present in Full1–Full4 cotyledon cells. The blank-nut phenotype has heavier nut shells. Overall, we conclude that single-nucleotide variants of *Acetyl-CoA carboxylase 1* (*ACC1*), *intracellular sodium/hydrogen exchanger 2* (*NHX2*), *UDP-glycosyltransferase 74E2* (*UGT74E2*), *DEFECTIVE IN MERISTEM SILENCING 3* (*DMS3*), *DETOXIFICATION 43* (*FRD3*), and *SS4* may induce embryo abortion, leading to blank-nut formation. Our results will benefit future research on how the gain or loss of candidate genes influences seed development. Moreover, our study provides novel prospects for seedless cultivar development.

## Introduction

Hazel (*Corylus* spp., Betulaceae, Fagales) is an important wind-pollinated species. Its nutritious and delicious nut is an important raw material for several food processing industries (Amaral et al., 2006). In recent years, the cultivation area and yield of hazelnut in China have increased rapidly. Hazel-related industries in mountainous areas of Northeast China, a major hazel cultivation region, have a significant effect on the development of local economy. Currently, *Corylus heterophylla* and hybrid hazel (*C. heterophylla × C. avellana*) are the most important species cultivated in China. Among these, the cultivation area of *C. heterophylla* is more than 1.0 million hm (Liu et al., 2014a; Liu et al., 2014b), more than that of hybrid hazel (Liu et al., 2014b; Cheng et al., 2018a; Cheng et al., 2018b). Thus, *C. heterophylla* contributes to most of the hazel products available in Chinese market.

Successful pollination, fertilization, and kernel development are prerequisites for good hazelnut yield. The failure of these important biological events during flower and fruit development might induce the drop of pistillate flowers, blank fruits, and shriveled kernels, causing varying degrees of yields loss. Frequent blank-fruit formation has been observed in *C. avellana* and *C. heterophylla* (Silva et al, 1996; Beyhan and Marangoz, 2007; Liu et al., 2012). Embryo abortion, rather than failed pollination or fertilization, has been implicated in blank-fruit formation (Liu et al., 2012). Seed formation is a pivotal process in plant reproduction and seed dispersal, and hazelnut kernel quality has been characterized previously (Ferreira et al., 2009; Ferreira et al., 2010; Xu and Hanna, 2010; Solar and Stampar, 2011). Furthermore, a set of genes that might be involved in regulating blank-fruit formation has been identified though transcriptome analysis of abortive and developing ovules; these differentially expressed genes were significantly enriched in the following Kyoto Encyclopedia of Genes and Genomes (KEGG) pathways: metabolism, plant hormone signal transduction, and RNA transport (Cheng et al., 2015a; Cheng et al., 2017). These studies provide clues about the mechanisms regulating hazel fruit quality. However, whether or not blank-fruit formation is induced at the transcriptome level remains unclear. Furthermore, the key genes involved in embryo development require further genetic evidence and verification.

Forward genetic screens can help elucidate the biological mechanisms in model species. Their success relies on the feasibility of mutant gene isolation (Nordström et al., 2013). Identification of causal mutations typically begins with genetic mapping, followed by candidate gene sequencing and complementation studies based on genetic transformation. Whole-genome re-sequencing of two Italian tomato landraces revealed sequence variations in genes associated with stress tolerance, fruit quality, and long shelf-life traits, and elucidated the genetic and molecular bases of fruit metabolism and storability in tomato (Tranchida-Lombardo et al., 2017). Using whole-genome re-sequencing and homozygosity mapping, it was found that the *SHELL* gene is responsible for the tenera phenotype (thin-shelled) in both cultivated and wild palms in sub-Saharan Africa (Singh et al., 2013). Thus, advances in DNA sequencing technologies have transformed genetic mapping and gene identification.

Hazel has unique flower and fruit developmental characteristics. The ovary is absent when the female pistillate inflorescence blooms. After pollination, pollen germination, and pollen tube growth, several layers of early ovary-primordium cells begin to differentiate. Thereafter, the ovary, ovule, and embryo sac take shape successively. *Corylus heterophylla* blooms in early- or mid-April in northeast China, and requires approximately two months from pollination to complete fertilization (Liu et al., 2014a). Subsequently, two ovules in the ovary begin to grow rapidly, but blank nuts form if embryo abortion occurs at this stage (Liu et al., 2012). In four artificial hazel orchards modified from natural forest, we found four mutant hazelnut trees that have apparently been producing only blank nuts for years. Their unique germplasm with the blank-nut phenotype could be valuable for mining key genes implicated in kernel development. In the present study, we therefore aimed to determine the genes involved in hazel embryo abortion, thus providing genetic and molecular bases for this process. The results should also promote the development of seedless germplasm in other fruit species.

## Materials and Methods

### Plant Materials

**Figure 1:**
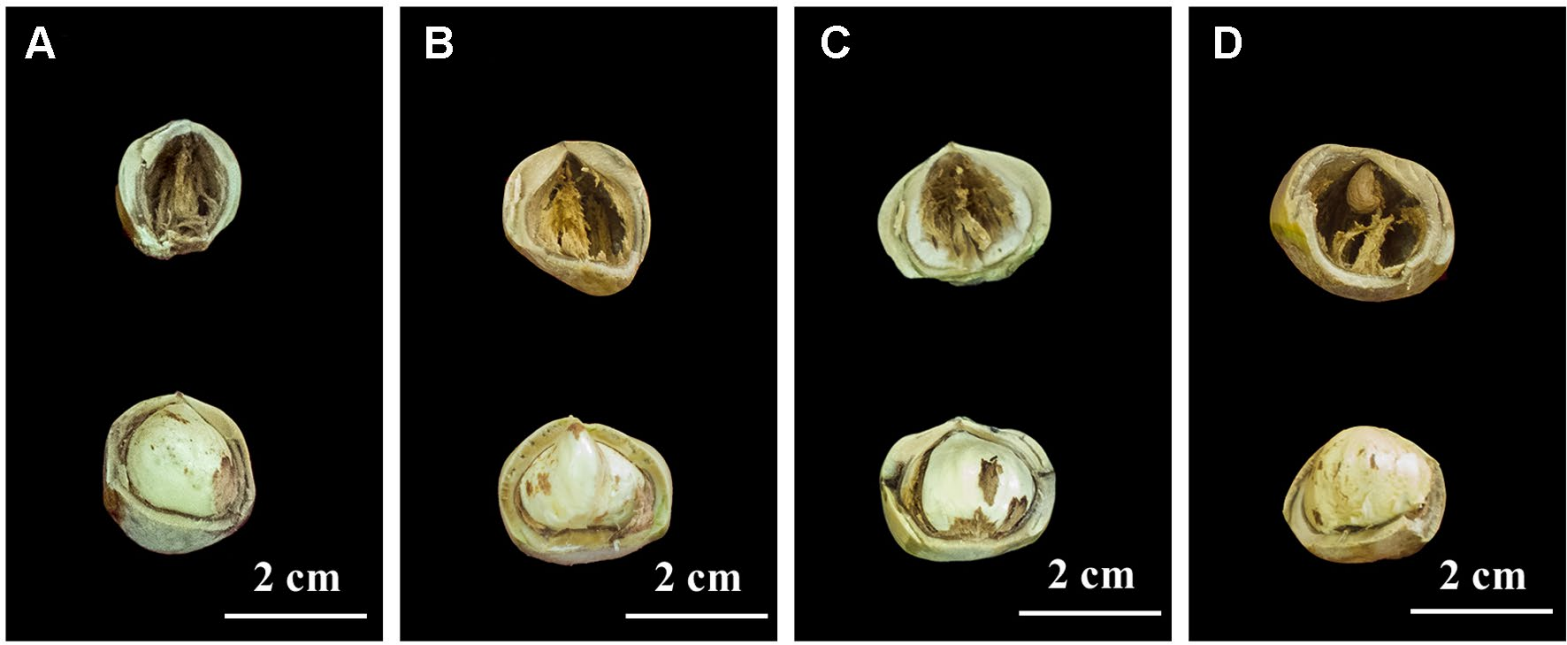
Nut characters of the eight hazel (*Corylus heterophylla*) trees sampled in northeastern China. **(A)** Empty1 (the first line) with blank nut trait and Full1 (the second line) with edible seed from Siping; **(B)** Empty2 (the first line) with blank nut trait and Full2 (the second line) with edible seed from Tieling; **(C)** Empty3 (the first line) with blank nut trait and Full3 (the second line)with edible seed from Kaiyuan; **(D)** Empty4 (the first line) with blank-nut trait and Full4 (the second line) with edible seed from Huludao.

In 2017, we found four hazel mutants that had borne only blank nuts for several years, in Northeast China. These mutant plants were distributed in four artificial orchards modified from hazel nature forest, in four different locations, namely Siping, Tieling, Kaiyuan, and Huludao Cities. In the orchard in Siping City, one plant bearing normal edible nuts, and another that had borne only blank nuts for several years, were selected; they were named Full1 and Empty1, respectively. To make the genetic background as close as possible, the spatial distance between the Empty1 plant and reference Full1 was less than 3.0 m. Similarly, Full2 and Empty2 (mutant) from Tieling City, Full3 and Empty3 (mutant) from Kaiyuan City, and Full4 and Empty4 (mutant) from Huludao City were sampled and analyzed. Blank nuts contain only small and unformed ovules, with no edible content (**Figure 1**). The pollen of several *Corylus heterophylla* × *C. avellana* cultivars (“Dawei,” “Bokehong,” and “Yuzhui”) and of *C. heterophylla* was collected and equally mixed following the method of Liu et al. (2014b). Primary artificial pollination was carried out, and the nut quality-analysis results confirmed that the blank-nut ratios were 100% for the four mutants, and that blank-nut formation in these mutants could not be relieved by artificial pollination. These chosen plants were subjected to molecular analysis to confirm their identity as *C. heterophylla*, using a simple sequence repeat (SSR)-based technique at Jilin Normal University. Seven primer pairs were used, according to the method of Cheng et al. (2018a).

In 2018, young leaves of the eight *C. heterophylla* plants mentioned above were collected and stored in liquid nitrogen for whole-genome re-sequencing. Meanwhile, some nut quality indexes were investigated, including shell weight, kernel weight, nut total weight, kernel ratio (kernel weight × 100/nut total weight), diameter, and blank-nut ratio.

### Isolation and Sequencing of DNA

DNA was isolated using the DNeasy Plant Mini Kit (QIAGEN, Valencia, CA, USA) according to manufacturer’s recommendations. DNA quality was evaluated by electrophoresis on a 0.8% agarose gel, and analyzed using a Nanodrop spectrophotometer (Thermo Fisher Scientific, Wilmington, USA) and Qubit 2.0 Fluorometer (Thermo Fisher Scientific Inc., Waltham, MA, USA). Genomic DNA was fragmented randomly using ultrasound. Fragments ranging from 200 to 500 bp in length were recycled by electrophoresis, and were connected with adaptors to generate clusters for sequencing. Eight paired-end fragment libraries averaging 300 bp were generated and sequenced on an Illumina HiSeq 2000 platform (Biomarker Technologies, Beijing, China) following Illumina protocols. The raw data were submitted to the National Center for Biotechnology Information (NCBI) (https://www.ncbi.nlm.nih.gov/Traces/study) (Accession number: PRJNA529018).

### Filtering and Assembling of Sequencing Data

The sequenced raw reads were filtered to obtain clean reads for further analysis. The following reads were removed: (1) paired reads with adaptor sequences; (2) paired reads with more than 10% unknown bases (N); (3) paired reads with a base quality value less than Q20; (4) paired reads shorter than 25 bp in length; and (5) paired reads with other than A, T, C, or G at the 5′ end. The filtered sequences were aligned to the reference Jefferson hazelnut genome (https://www.cavellanagenomeportal.com/) to detect the genomic mutations in our samples using Burrows–Wheeler Aligner software (Li and Durbin, 2009). Picard tools (https://broadinstitute.github.io/picard/) were used to remove duplicate reads, and the BAM file generated was used to calculate sequencing depth and coverage. Genome Analysis Toolkit (GATK) and SAMtools were used to map the obtained genome sequence data to the reference hazelnut genome and call high-quality SNPs (Li et al., 2009; Van der Auwera et al., 2013). GATK software was used to locally re-align reads near indels and generate BAM files after realignment; this can eliminate false positive SNPs around indels. Then, GATK was used to detect SNP, and filter out the sites with lower sequence depth and quality score. The genotype information of each point was obtained in the final VCF file. Based on the comparison results of GATK, VarScan 2 software (version 2.4.1) was used to obtain information about SNPs (Koboldt et al., 2009) and remove variable sites with relatively low sequencing depth and quality, to obtain a highly reliable data set. Filtering parameters were as follows: lowest coverage depth, 30 reads; minimum mutation frequency, 20%; minimum mutation base number, 15 reads; lowest quality value for mutant sites, Q20; and both positive and reverse reads supported the mutation site, and the difference between the number of positive and reverse reads was less than 10%. ANNOVAR software was used to obtain SNP annotations (Wang et al., 2010). Based on the SNP information for each sample, online software PHYLIP version 3.697 (http://evolution.genetics.washington.edu/phylip.html) was used to construct the phylogenetic tree of the eight hazel samples, using neighbor-joining method (**Figure S1**).

### Identification of Major Genes Regulating Embryo Abortion

To narrow the range of candidate genes, several analyses were performed in succession on detected SNPs and their positional data. First, online Venn Diagram tools (http://bioinformatics.psb.ugent.be/webtools/Venn/) were used to make SNP-paired comparisons of Empty1 vs. Full1, Empty2 vs. Full2, Empty3 vs. Full3, and Empty4 vs. Full4, thereby identifying the SNPs unique to the blank-nut mutants. A common intersection of SNPs was also generated from the comparisons. Candidate genes with common and unique SNPs in their exon regions were selected for Gene Ontology (GO) analysis using WEGO 2.0 (http://wego.genomics.org.cn/). Genes associated with embryo development were considered to be those involved in transporter activity function (Liu et al., 2013), and those with homozygous SNPs in Empty1–4. Finally, cis elements in the *starch synthase 4* (*SS4*) promoter (a 2 000 bp region upstream of the start codon) were analyzed using PlantCARE (http://bioinformatics.psb.ugent.be/webtools/plantcare/html/).

### Validation of SNP Assays

Genomic DNA from each sample was PCR-amplified. Three primer pairs designed using the reference sequence of *SS4* (*g16468*) (forward primer: 5′-TGTTGAAAAGACTGTCGCTGAGAAG; reverse primer: 5′-CTTCCATCTCTCTTCCACACCATTT), *acetyl-CoA carboxylase 1* (*ACC1*, *g11831*) (forward primer: 5′-TTTGGACTCTAATATTGCTGAAG; reverse primer: 5′-TAAGGTAGATCAGCCATGCAGTA), *sodium/hydrogen exchanger 2* (*NHX2*, *g12219*) (forward primer: 5′-TGGTGGAAGAAGGTATATAAAGAA; reverse primer: 5′-GGTTCTTTGTTCACATCTCTTTAA). Approximately 30 ng of genomic DNA was amplified using a Bio-Rad thermal cycler (Bio-Rad, Hercules, CA). The reaction volume was 20 µl, comprising 1.0 µl genomic DNA template, 0.4 µM primers, and 10.0 µl 2 × Taq plus Master Mix (Tiangen Biotech Co., Ltd., Beijing, China). The thermocycling protocol was as follows: 95°C for 5 min, followed by 35 cycles of 95°C for 30 s, 55°C for 20 s, and 72°C for 60°s. Amplicons were subsequently cloned into the pMD19-T vector (Takara), transformed into *Escherichia coli* DH5a cells following manufacturer protocol, and sequenced by Sangon Biotech Co., Ltd (Shanghai, China).

### Cytological Analysis of Starch Granules in Cotyledons

The cotyledons were isolated from the seeds under a stereoscope, and cut into cubes of edge length 1–2 mm. These cubes were fixed in 4% polyformaldehyde solution for 24 h and stored in 70% ethanol at −20°C until further use. The samples were then dehydrated in gradient ethanol solutions. Dehydration, embedding, and aggregation were performed using the LR White Resin Embedding Kit (Electron Microscopy Sciences, USA), following the protocol of the manufacturer; 0.5% periodic acid and Schiff reagent were used to stain starch granules in the cotyledons. Cytogenetic observation of starch granules in the sections was performed and photographed using optical light microscopy.

## Results

### Overview of Sequencing Data

Whole-genome re-sequencing of our eight samples generated 42.01 Gb of clean data; statistical analysis results are shown in **Table 1**. These data were aligned to the 289.80-Mb reference genome. The sequencing depth ranged from 16.38× to 25.03×, covering 67%–73% of the reference genome bases. The Illumina sequence data were assembled using software SPAdes 3.0 (Bankevich et al., 2012), and 97.9% of the assembled sequences, on average, could be mapped to the reference genome. This indicates that the *C. heterophylla* samples have a close genetic relationship with *C.* avellana.The base error rate was lower than 0.02%. The percentage of bases with Phred value >20 was more than 94%. The GC count per read followed a normal distribution. These results indicate that library construction and quality by Illumina sequencing were reliable.

**Table 1:** Statistical analysis results of clean sequencing data, for eight hazel (*Corylus heterophylla*) trees sampled in Northeast China.

Sample	Total reads	Total bases	Error %	Q20%	Q30%	GC %	Depth	Coverage
Empty1	32685090	4807907701	0.0183	95.01	88.72	38.94	15.82	0.71
Empty2	32862042	4792174301	0.0184	94.92	88.62	37.26	15.77	0.73
Empty3	39550994	5744594343	0.0187	94.78	88.34	37.67	18.90	0.67
Empty4	40402514	5925325425	0.0156	96.20	91.20	38.87	19.50	0.71
Full1	39614452	5725619379	0.0186	94.79	88.37	38.39	18.84	0.70
Full2	38213274	5537932084	0.0183	94.96	88.71	37.52	18.22	0.71
Full3	33925356	4978413145	0.0192	94.62	87.91	43.02	16.38	0.71
Full4	51797148	7606639739	0.0152	96.30	91.58	38.88	25.03	0.70

Error %, Base error rate. Q20 and Q30, the percentage of bases with the Phred value > 20 and > 30, respectively. GC %, the percentage of G and C bases. Depth, sequencing depth; Coverage, coverage ratio, obtained base number relative to the total base number of reference hazelnut genome.

### Single-Nucleotide Polymorphism Detection

Mapping of our genome sequencing data to the reference hazelnut genome using GATK was performed to call high-quality SNPs. In the samples of Empty1, Empty2, Empty3, and Empty4, 1 494 330, 1 539 833, 2 065 212, and 214 6447 SNPs were identified, respectively (**Table 2**); in the samples of Full1, Full2, Full3, and Full4, 1 982 873, 1 979 751, 965 214, and 2 775 577 SNPs were identified, respectively (**Table 2**). In total, 3 391 427 SNPs were identified using our eight samples. The distribution of SNPs showed an notable preference, and the ratio of SNPs located in the intergenic region was the highest (43%–47%), followed by that in the exonic (14%–24%), intronic (16%–19%), upstream (7%–9%), and downstream regions (6%–8%) (**Table 2**). The SNP mutation sites in the exon regions might have potential effects on protein translation. Approximately 52% of the SNP mutations in the exon regions can lead to nonsynonymous mutation at the translation level (**Table 3**).

**Table 2:** Statistical analysis results of SNP annotation, for eight hazel (*Corylus heterophylla*) trees sampled in Northeast China.

Location	Empty1	Empty2	Empty3	Empty4	Full1	Full2	Full3	Full4
Downstream	104704	116897	161416	162810	155144	154536	60405	212824
Exonic	283283	230763	296316	353058	315313	292243	223867	409446
Intergenic	647664	713970	969730	946126	876796	912011	398024	1290521
Intronic	258128	286216	375095	399487	378273	366754	155008	488644
Splicing	1588	1252	1677	2055	1859	1595	1261	2592
Upstream	117029	113107	157114	165578	149542	151003	71747	225400
Upstream; downstream	8034	8341	11881	12793	11979	11523	4797	16430
UTR3	44169	45400	60344	67548	61792	59537	28643	82809
UTR5	29731	23887	31639	36992	32175	30549	21462	46911
Total	1494330	1539833	2065212	2146447	1982873	1979751	965214	2775577

**Table 3:** Statistical analysis results of the effect of SNP mutation sites in the exon regions on protein translation, for eight hazel (*Corylus heterophylla*) trees sampled in Northeast China.

	Empty1	Empty2	Empty3	Empty4	Full1	Full2	Full3	Full4
Nonsynonymous SNV	146644	120223	155071	185796	164205	151959	116828	216738
Stopgain SNV	4205	3273	4413	5504	4759	4166	3263	6636
Stoploss SNV	674	568	726	874	737	715	560	1117
Synonymous SNV	116226	94620	120275	141946	128290	119902	91310	161742
Unknown	15534	12079	15831	18938	17322	15501	11906	23213

SNV, single-nucleotide variant.

### Unique and Common SNPs in the Blank-Nut Mutants

**Figure 2:**
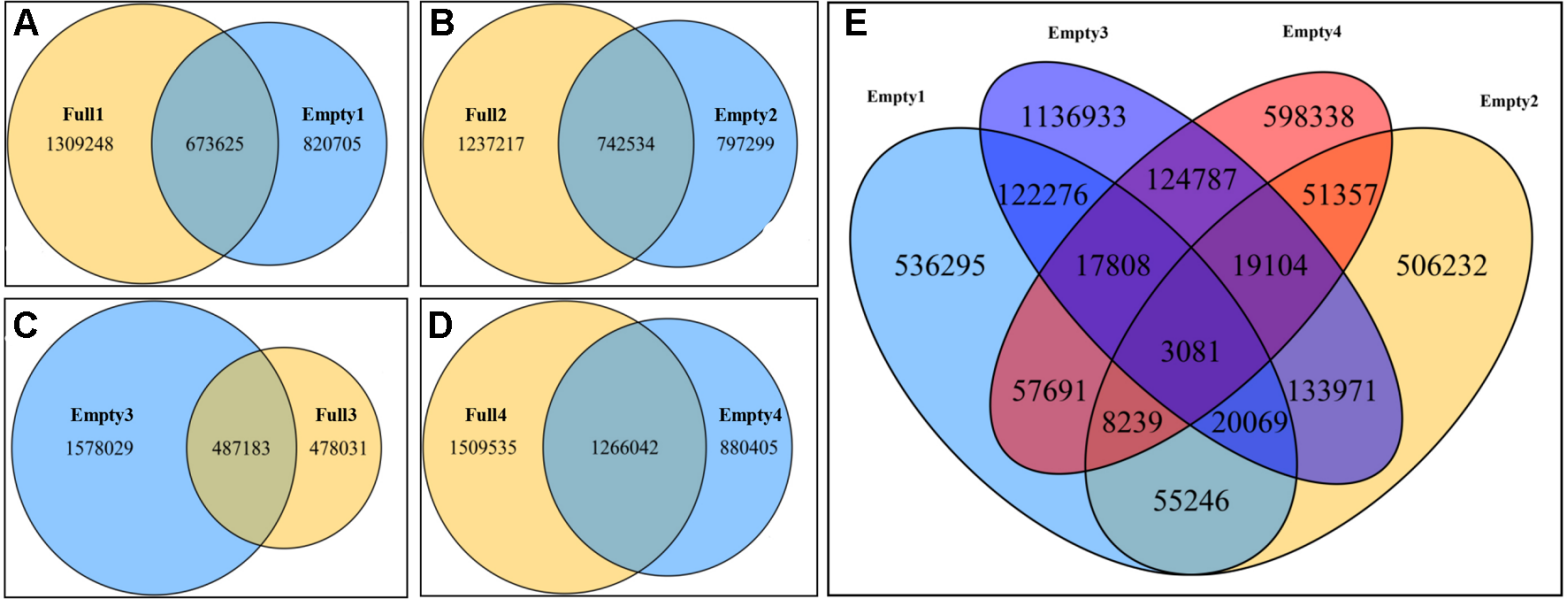
Common and unique single-nucleotide polymorphisms (SNPs) in blank-nut mutants of hazel (*Corylus heterophylla*) sampled in Northeast China. **(A)** Paired comparison of Full1 vs. Empty1; 82 075 unique SNPs were found in the blank-nut mutant Empty1; **(B)** paired comparison of Full2 vs. Empty2; 797 299 unique SNPs were found in the blank-nut mutant Empty2; **(C)** paired comparison of Full3 vs. Empty3; 478 031 unique SNPs were found in the blank-nut mutant Empty3; **(D)** paired comparison of Full3 vs. Empty3; 880 405 unique SNPs were found in the blank-nut mutant Empty4; **(E)** unique SNPs in the blank-nut mutants revealed using Venn Diagrams.

Paired comparisons of SNPs between two samples from the same orchard were carried out to identify unique SNPs in the blank-nut mutants using Venn Diagrams tools. Consequently, 820 705, 797 299, 1 578 029, and 880 405 were identified by paired comparison of Empty1 vs. Full1, Empty2 vs. Full2, Empty3 vs. Full3, and Empty4 vs. Full4, respectively (**Figures 2A–D**). Based on these findings, common and unique SNPs in the blank-nut mutants were identified, using the four groups of SNPs mentioned above. In total, 3 081 common and unique SNPs were detected using the Venn Diagrams tools (**Figure 2E**). For SNP mutations to occur in the exon regions, the nonsynonymous single-nucleotide variants (SNVs) should induce changes in amino acids at the protein translation level. Among the 3,081 common and unique SNPs (**Table S1**), 215 SNPs belonged to nonsynonymous SNVs in the exon region, and they were distributed among 178 candidate genes (**Table S2**).

### Analysis of SNP Heterozygosity and Validation of SNP Assays

**Figure 3:**
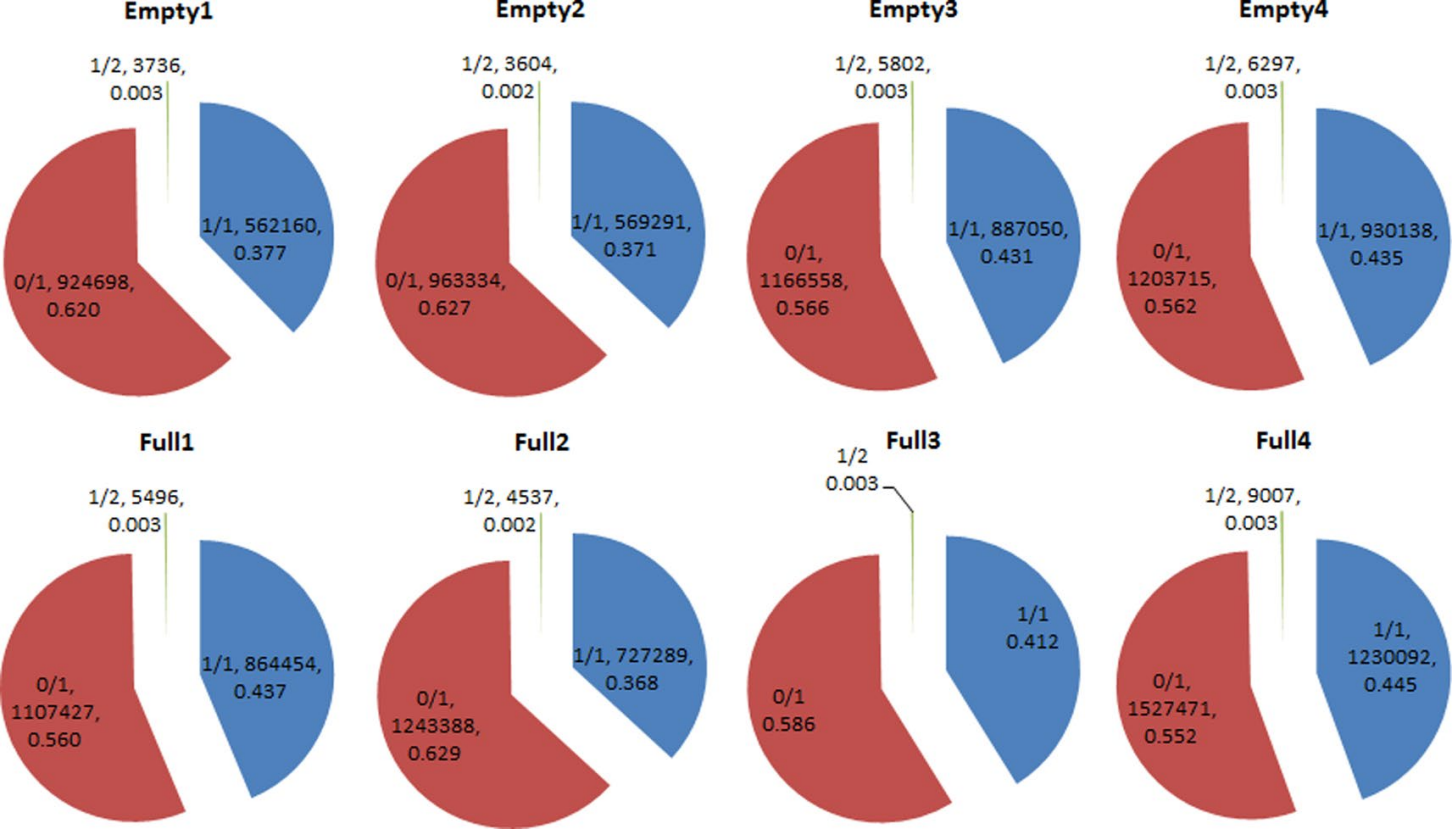
Heterozygosity statistical results of single-nucleotide polymorphisms (SNPs) in hazel (*Corylus heterophylla*) trees sampled in Northeast China. 1/1: homozygous genotype; 0/1 and 1/2: heterozygous genotypes. The pie charts each show three parts representing three genotypes, and three parameters that represent the genotype of SNP, the number of SNPs, and the corresponding percentage of SNPs, respectively.

We identified 14 908 159 SNPs in all eight samples. Among these samples, homozygous (1/1) SNP ratios ranged from 0.377 to 0.445, while heterozygous (0/1) SNP ratios ranged from 0.552 to 0.629. The 1/2 heterozygous genotype was scarce, with a ratio of <0.004 in all samples. Average homozygous and heterozygous SNP ratios were 0.409 and 0.591, respectively (**Figure 3**). Among the 178 candidate genes (**Table S3**), 18 had the homozygous SNP genotype in Empty1, Empty2, Empty3, and Empty4 (**Table S4**). Three genes of interest were selected for PCR validation: starch synthase 4 (*SS4*, g16468), Acetyl-CoA carboxylase 1 (*ACC1*, g11831), and sodium/hydrogen exchanger 2 (*NHX2*, g12219). The results were consistent with those from whole-genome re-sequencing.

### Gene Ontology Analysis of Candidate Genes

**Figure 4:**
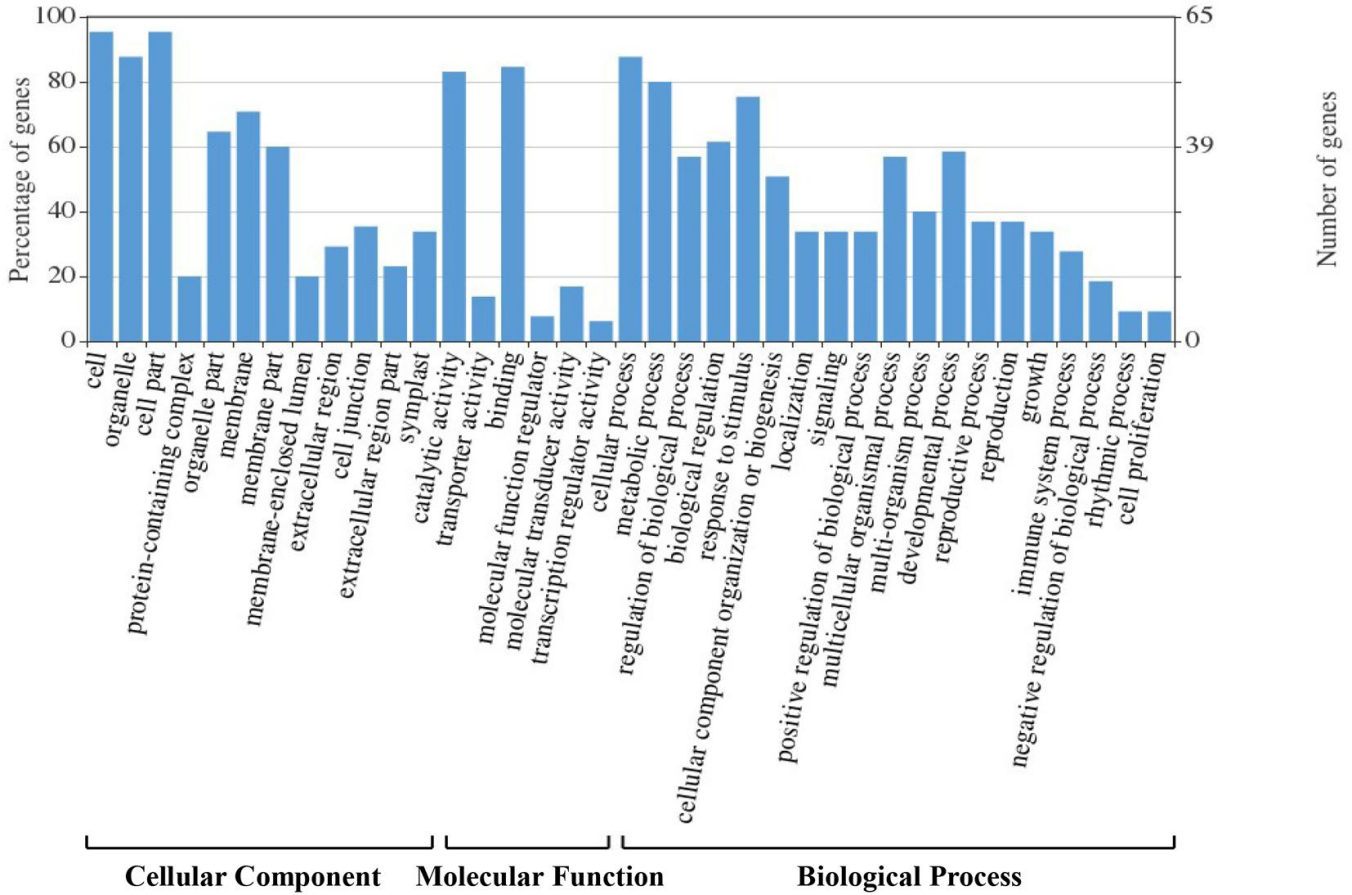
Gene Ontology (GO) classification analysis of the 178 candidate genes identified in hazel (*Corylus heterophylla*) sampled in Northeast China.

We assigned 178 candidate genes to the following three GO categories: biological processes, cellular components, and molecular functions (**Figure 4**). The majority of these genes were involved in binding, catalysis, molecular transducers, transporters, and molecular function regulators. They were also enriched in the categories cellular and metabolic processes, response to stimulus, developmental process, regulation of biological process, multicellular organismal process, cellular component organization or biogenesis, multi-organism process, reproductive process, localization, signaling, and positive regulation of biological process. Most corresponding proteins were located in organelles and the cell membrane.

### *Cis* Elements in the Gene Promoter

The nine genes involved in transporter molecular functions according to GO analysis were chosen for further cis element analysis. These genes were thought to be involved in regulating embryo abortion and are expected to be expressed in seeds. Only *SS4* (g16468) had the RY-element indicative of seed-specific regulation, however (**Table 4**). In the SS4 promoter region, we found other cis elements including ABRE, Box 4, G-Box, GARE-motif, and P-box, suggesting that abscisic acid, light, and gibberellin regulate SS4 expression.

**Table 4:** Cis element in the promoter region of *SS4*.

Cis element	Organism	Location /DNA	Matrix score	Sequence	Biological function
ABRE	*A. thaliana*	1843/+	5	ACGTG	Cis-acting element involved in the abscisic acid responsiveness
Box 4	*P. crispum*	429/+	6	ATTAAT	Part of a conserved DNA module involved in light responsiveness
	*P. crispum*	1450/-	6	ATTAAT	
	*P. crispum*	767/+	6	ATTAAT	
G-Box	*P. sativum*	1842/-	6	CACGTT	Cis-acting regulatory element involved in light responsiveness
GARE-motif	*B. oleracea*	579/-	7	TCTGTTG	Gibberellin-responsive element
GATA-motif	*A. thaliana*	401/+	7	GATAGGA	Part of a light responsive element
	*A. thaliana*	466/-	10	AAGATAAGATT	
P-box	*O. sativa*	1052/+	7	CCTTTTG	Gibberellin-responsive element
RY-element	*H. annuus*	1040/-	8	CATGCATG	Cis-acting regulatory element involved in seed-specific regulation
TATC-box	*O. sativa*	230/+	7	TATCCCA	Cis-acting element involved in gibberellin-responsiveness
	*O. sativa*	1639/-	7	TATCCCA	
	*O. sativa*	1361/+	7	TATCCCA	
TCT-motif	*A. thaliana*	742/-	6	TCTTAC	Part of a light responsive element

### Single-Nucleotide Variant in SS4

**Figure 5:**
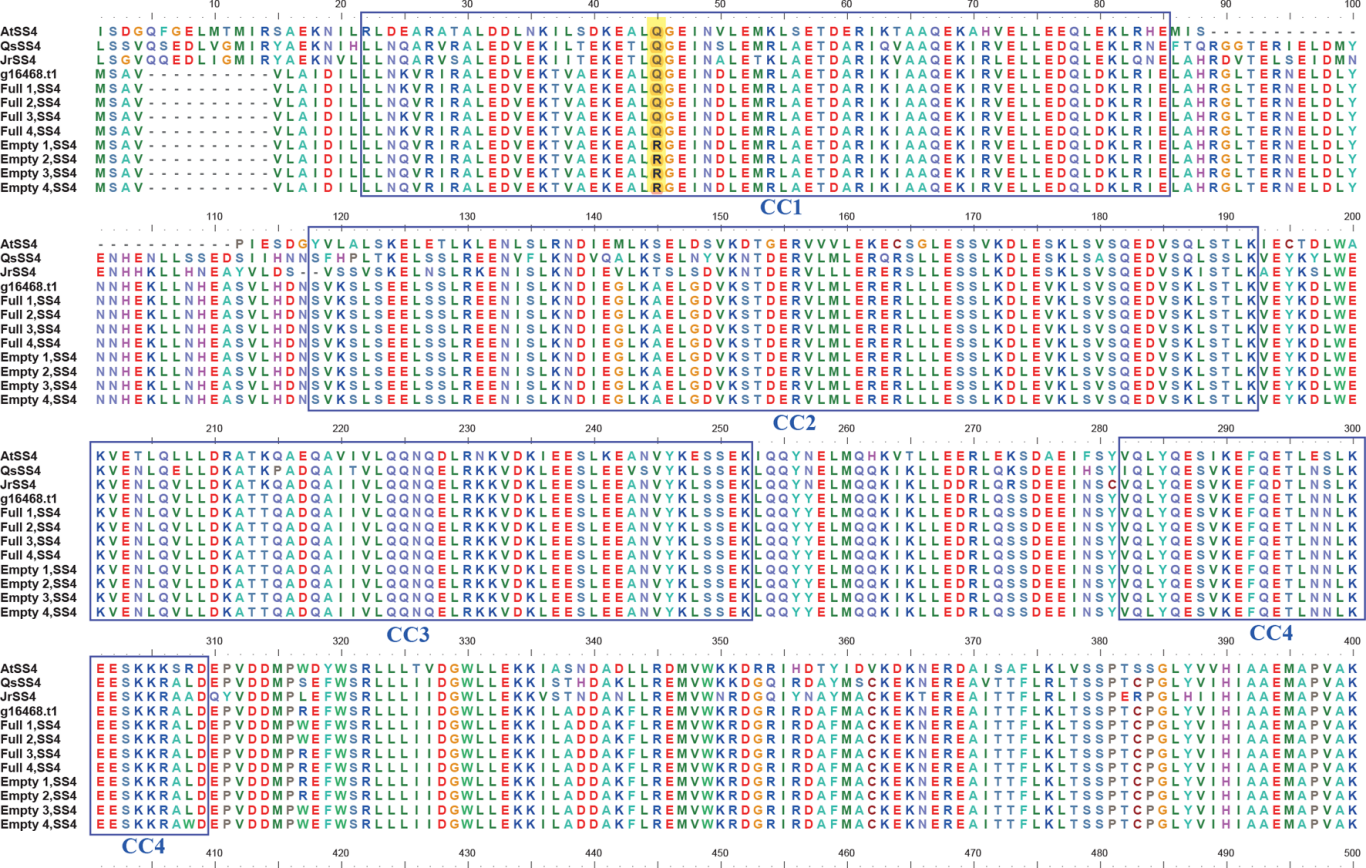
Comparison of the amino acid sequence of starch synthase 4 (SS4) in hazel (*Corylus heterophylla*) sampled in northeastern China with SS4 from other plants. *AtSS4*, *Arabidopsis thaliana SS4*; *QsSS4*, *Quercus suber SS4*; *JrSS4*, *Juglans regia SS4*; *g16468.t1, Corylus avellana SS4*; Empty, blank-nut mutant; Full, edible seed.

BLASTP searches of the eight SS4 genes were performed against a protein database containing the model plant *Arabidopsis thaliana* and related species, such as *Corylus avellana*, *Quercus suber*, and *Juglans regia*. The results identified four coiled-coil domains at the N terminal of SS4. Additionally, the SNV of the blank-nut mutants occured in the CC1 region (**Figure 5**). The sequence from the 40^th^ to 49^th^ amino acid in the CC1 region was highly conserved in all tested species. In the four blank-nut mutants, the 45^th^ amino acid residue mutated from a hydrophilic glutamine (Q) to an alkaline arginine (R). At the same position, Full1–4 maintained a high degree of similarity with related sequences in *C. avellana*, *Q. suber*, and *J. regia*. The results of SS4 SNP heterozygosity analysis show that Empty1, 2, and 4 were heterozygous (0/1), while Empty3 was homozygous (1/1) at this SNV loci (**Table S3**).

### Observation of Starch Granules in Cotyledons

**Figure 6:**
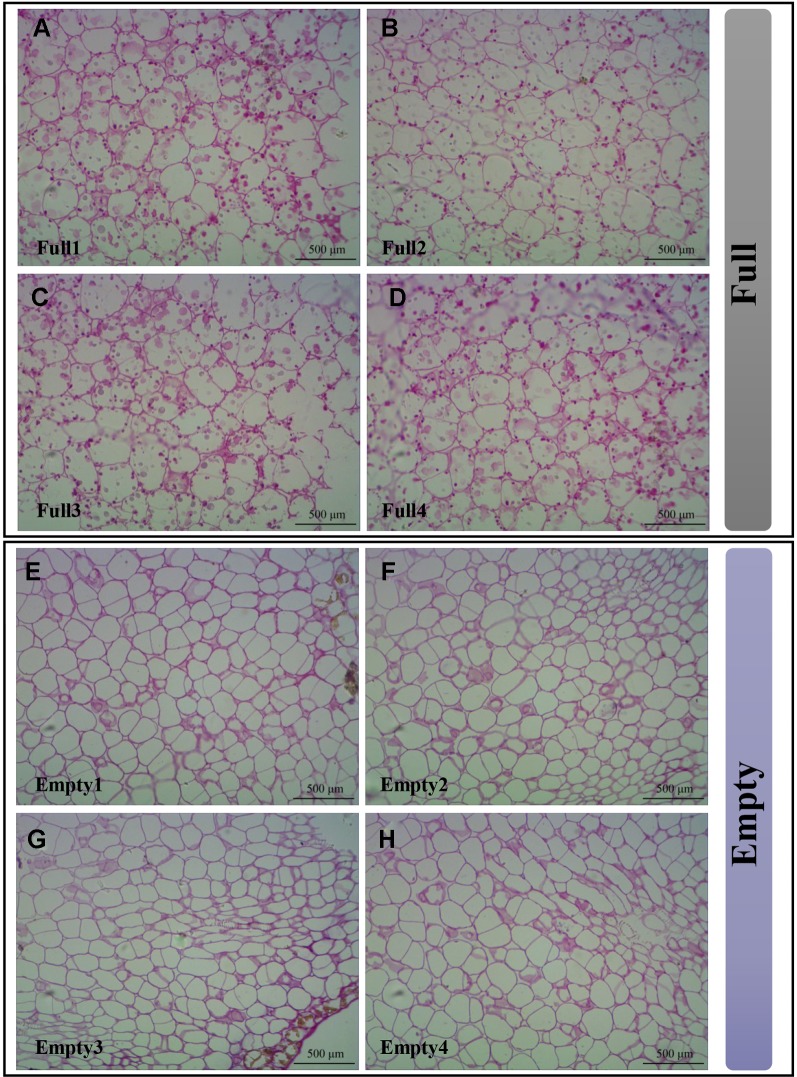
Starch granules observed in cotyledons of hazel (*Corylus heterophylla*) sampled in Northeast China. **(A)** Full1; **(B)** Full2; **(C)** Full3; **(D)** Full4; **(E)** Empty1; **(F)** Empty2; **(G)** Empty3; **(H)** Empty4. Empty, blank-nut mutant; Full, edible seed.

To demonstrate that the SS4 mutation causes embryo abortion, we used resin sections stained with periodic acid and Schiff reagent to analyze starch granule distribution in cotyledons. We observed multiple starch granules in all cotyledon cells of Full1–4 (**Figures 6A–D**). However, Empty1–4 cotyledons were nearly devoid of starch granules (**Figures 6E–H**). These results strongly suggest that an SNV of *SS4* impaired starch biosynthesis in Empty1–4 embryos.

### Statistical Analysis of Nut Characteristics

We found clear differences in blank versus wild-type nut characteristics from plants of the same orchard. Mutants had significantly higher nutshell weight, along with significantly lighter kernel and total nut weight. Consequently, mutant nuts exhibited a greater shell ratio and smaller kernel ratio (**Table 5**). These results suggested that photosynthate transport to nuts occurred preferentially in the shell rather than the kernel, leading to heavier shell weight among blank nuts. However, blank and wild-type nuts did not differ in diameter (**Table 5**). Occasionally, Full1 and Full 2 produced blank nuts, while the four mutants produced 100% blanks without exception (**Table 5**).

**Table 5:** Characteristics of nuts used in the present study, from eight hazel (*Corylus heterophyll* *a*) trees sampled in northeastern China.

Sample	Shell Weight (g)	Kernel Weight (g)	Total nut weight (g)	Shell ratio (%)	Kernel ratio (%)	Diameter (cm)	Blank-nut ratio (%)
Empty1	1.215 b	0.003 e	1.218 cd	99.754 a	0.246 c	2.40 ab	100 a
Full1	1.091 c	0.642 a	1.733 a	62.954 c	37.046 a	2.44 ab	2.47 c
Empty2	1.148 bc	0.003 e	1.151 d	99.739 a	0.261 c	2.29 b	100 a
Full2	0.964 d	0.573 b	1.537 b	62.720 c	37.280 a	2.34 ab	3.33 b
Empty3	1.32 a	0.003 e	1.323 c	99.773 a	0.227 c	2.53 a	100 a
Full3	1.181bc	0.526 c	1.707 a	69.186 b	30.814 b	2.52 a	0 d
Empty4	0.942 d	0.003 e	0.945 e	99.683 a	0.317 c	1.89 c	100 a
Full4	0.799 e	0.358 d	1.157 d	69.058 b	30.942 b	1.94 c	0 d

Values within the same column followed by different lowercase letters are significantly different at P ≤ 0.05 as indicated by the LSD test. A nut with less than 5% kernel ratio is defined as a blank nut.

## Discussion

*Corylus heterophylla* (family Betulaceae) is unique to China and the major source of hazelnut products in China (Liu et al., 2014b). We currently have little genetic information about this important agricultural product. Here, our assembled genomic sequences, mapped to the reference Jefferson hazelnut (*C.* avellana) genome, revealed that the two plants are closely related. Thus, the *C.* avellana genome information can also be used for *C. heterophylla* gene identification and mining. In Northeast China, many artificial hazel orchards have been created by modifying natural *C. heterophylla* forests, for the purposes of protecting forests. Compared with the nuts of *C. heterophylla × C. avellana* cultivars, those of *C. heterophylla* possess thicker shells, lower kernel ratio, higher blank-nut ratio, and poorer nut quality. However, *C. heterophylla* has exceptional cold resistance and thus the potential to be cultivated in more regions. Therefore, this was an unprecedented opportunity to identify important genes regulating kernel development, using our four blank-nut mutant germplasms. Evolutionary tree analysis based on obtained SNP information showed that Full2, Empty2, Full3, and Empty3 are on four different genetic branches (**Figure S1**), indicating that sexual propagation may play an important role in *C. heterophylla* population formation, despite the common use of tiller propagation.

*Corylus heterophylla* is an anemophilous species with pollen self-incompatibility and a heterozygous genome (2n = 22). Consistent with this, we found that most SNPs were heterozygous. The fact that 37.7–44.5% of SNPs were homozygous implies that selfing likely plays an important role in hazelnut population formation and evolution.

### Important Candidate Genes That May Regulate Embryo Abortion

In the present study, paired comparisons based on whole-genome re-sequencing and SNP screening revealed 3,081 common SNPs in Empty1, Empty2, Empty3, and Empty4. Among these, 215 nonsynonymous SNPs, distributed across 178 candidate genes, appear to induce amino acid changes at the translation level, while 18 genes had the 1/1 homozygous SNP genotype. Homozygous SNP mutation in *C. heterophylla* indicates simultaneous base mutation in both alleles, which appears more likely than heterozygous SNP mutation to induce observable phenotypes. After filtering unannotated genes, integrated biological functions, and embryo-abortion characteristics, we identified a set of gene products implicated in regulating blank-nut formation, including ACC1, NHX2, UDP-glycosyltransferase 74E2 (UGT74E2), DEFECTIVE IN MERISTEM SILENCING 3 (DMS3), and DETOXIFICATION 43 (FRD3) (**Table 6**). We discuss each of these proteins briefly below.

**Table 6:** Interest common, unique, and nonsynonymous SNPs in coding regions of Empty1, Empty2, Empty3, and Empty4.

No.	CHROM	POS	REF	ALT	HE-HO	Gene Name	Gene Annot
1	00025	108257	G	T	1/1	g526	Protein DEFECTIVE IN MERISTEM SILENCING 3, DMS3
2	00355	58664	G	C	1/1	g3976	Protein DETOXIFICATION 43, DTX43 or FRD3
3	00852	15014	C	T	1/1	g7423	Probable xyloglucan glycosyltransferase 5, CSLC5
4	01112	39903	G	A	1/1	g8887	O-fucosyltransferase 34, OFUT34
5	01581	4652	C	T	1/1	g11171	Probable terpene synthase 9, TPS9
6	01736	26257	G	A	1/1	g11831	Acetyl-CoA carboxylase 1, ACC1
7	01830	22598	C	A	1/1	g12219	Sodium/hydrogen exchanger 2, NHX2
8	02132	19138	A	G	1/1	g13442	Non-functional pseudokinase ZED1, ZED1
9	05024	10274	G	C	1/1	g21444	Ankyrin repeat-containing protein BDA1, BAD1
10	07995	10257	C	A	1/1	g26083	U-box domain-containing protein 34, PUB34
11	08504	7843	T	A	1/1	g26696	Disease resistance protein At4g27190, At4g27190
12	08703	4254	A	T	1/1	g26905	UDP-glycosyltransferase 74E2, UGT74E2
13	03041	13510	A	G	0/1, 1/1	g16468	Starch synthase 4, SS4

All the SNP mutation types in coding regions are exonic and nonsynonymous; Chrom, reference sequence name; Abbreviation of reference sequence name was used. Eg. C.avellana_Jefferson_00025 was abbreviated as 00025. POS, SNPs (single-nucleotide polymorphisms) position on reference sequences. Ref, Reference base of SNP; ALT, Variant bases of SNP; HE-HO, Homozygous and heterozygous genotype of SNP. The homozygous genotype was 1/1, and the heterozygous genotype was 0/1, 1/2; Gene Annot, Annotation of gene with singl- nucleotide variant.

Mature hazelnut is rich in unsaturated fatty acids. The homozygous SNP mutation of *ACC1* may impair citrate conversion to long-chain fatty acids and trigger physiological disorders in ovules. Furthermore, *ACC1* loss-of-function led to abnormal embryo morphogenesis and embryo lethality in transgenic *Arabidopsis* seeds (Baud et al., 2003).

NHX2 has vital effects on cellular pH and Na^+^/K^+^ homeostasis. The double-knockout mutants of *nhx1/nhx2* caused significantly reduced growth, smaller cells, and shorter hypocotyls in etiolated seedlings, as well as abnormal stamens in mature flowers (Bassil et al., 2011).

UDP-glycosyltransferase 74E2 (UGT74E2) is an auxin glycosyltransferase. In *Arabidopsis*, *UGT74E2* overexpression disrupted indole-3-butyric acid and auxin homeostasis, thus altering plant architecture while improving stress tolerance (Tognetti et al., 2010). Immunohistochemical analysis showed that auxin distribution was in enriched at the growth center of ovaries during early ovule formation, implying that the enzyme is important in regulating ovule development (Cheng et al., 2018b).

DNA methylation is an epigenetic alteration related to gene silencing. In the RNA-directed DNA methylation (RdDM) pathway, DMS3 is required in producing Pol V-dependent transcripts, suggesting a regulatory role in DNA methylation and gene silencing (Law et al., 2010). The total methylation ratio of hazel ranges from 44.61 to 48.68% before pollination, but then decreases by ∼4% after pollination, suggesting that epigenetic changes are an important mechanism for initiating ovary and ovule development (Cheng et al., 2015b).

*FRD3* encodes a membrane protein belonging to the multidrug and toxin efflux family, which is involved in transporting small organic molecules. In *Arabidopsis*, *frd3* mutant plants were defective in either iron-deficiency signaling or iron distribution, indicating that FRD3 is an important component of iron homeostasis (Rogers and Guerinot, 2002).

Taken together, homozygous SNP mutations in *ACC1, NHX2, UGT74E2, DMS3*, and *FRD3* impair numerous important biological processes that would induce embryo abortion in ovules.

### *SS4* May Play an Important Role in Regulating Hazelnut Embryo Abortion

Starch is important in the metabolism of photosynthetic organisms, accumulating in chloroplasts during the day and degrading at night to supply energy for growth (Stitt and Zeeman, 2012). Over the long term, starch can also be stored in seed endosperm, tubers, and other storage organs, providing energy for plant growth, seed germination, and other biological processes (Toyosawa et al., 2016). Starch synthases are transglycosylases that elongate the α-1,4-glycoside bond through glycosyl transfer, using ADP as a glucose donor (Miura et al., 2018). SS4 specifically controls starch-grain abundance in the chloroplast. Deletion mutations of SS4 result in severely restrict starch grains per chloroplast. Moreover, the core of starch grains in the mutants also differed significantly from those of the wild type, indicating that SS4 participates in the initiation of starch-grain biosynthesis (Crumpton-Taylor et al., 2013; Raynaud et al., 2016). The N-terminal part of *Arabidopsis* SS4 (At4g18240) contains 543 amino acids, and the sequence is well conserved across the SS4 proteins of species sequenced to date (Raynaud et al., 2016). The N-terminal part of At4g18240 is divided into five segments: CC1 (coiled-coil domain 1), CC2, CC3, CC4, and CR (conserved region). The SS4 fragment containing CC1 and CC2 interacts with the FBN1b protein located on the plastoglobule surface, potentially facilitating SS4’s association with the thylakoid membrane (Gámez-Arjona et al., 2015). Deletion of a long coiled-coil region at the N terminus of SS4 (Roldán et al., 2010) prevents the enzyme from binding to fibrillin 1 and alters its localization in the chloroplast. Thus, SS4 localization and its interaction with fibrillins are mediated by the N-terminal segment (Gámez-Arjona et al., 2015). A rice (*Oryza sativa* L.) double-mutant *SSIIIa* and *SSIVb* (*ss3a ss4b*) generated spherical starch granules in seeds, whereas single mutants produced polyhedral starch granules similar to wild-type (Toyosawa et al., 2016). Overexpressing SSIV increased starch content in *Arabidopsis* leaves by 30% to 40%. For long-term storage of starch in potato tubers, SSIV overexpression increases tuber starch content and yield (Gámez-Arjona et al., 2011). Thus, SS4 is responsible for the initiation of starch-grain biosynthesis in both chloroplasts and storage organs, and its N-terminal determines localization in cells.

Our previous study indicated that photosynthates were not transported to abortive ovules in blank nuts (Liu et al., 2013). Our current findings imply that genes involved in transport could also regulate embryo abortion in hazelnut, and those involved in photosynthate transport might induce ovule developmental failure. Only the SS4 promoter region contained the RY-element, implying its seed-specific expression pattern. An SNV of SS4 showed that the 45^th^ amino acid residue in the highly conserved CC region of N-terminal mutates from a hydrophilic glutamine (Q) to an alkaline arginine (R) in Empty1, Empty2, Empty3, and Empty4, whereas this region in Full1, Full2, Full3, and Full4 was similar to those in *C. avellana*, *Q. suber*, and *J. regia*. An SNV of SS4 at the N terminal might impair appropriate protein localization and induce the failure of starch-grain biosynthesis. This hypothesis is consistent with previous data showing ovule abortion soon after fertilization (Liu et al., 2012). We also confirmed that starch granules were absent in cotyledon cells of Empty1–4 and present in cotyledon cells of Full1–4. Therefore, the starch content in abortive ovaries was significantly lower than in developing ovule.

Of the four blank-nut mutants, three were heterozygous and one was homozygous at the 45^th^ amino acid residue of the SS4 N-terminal. As it is diploid, mutation in one of paired chromosomes of *C. heterophylla* may be enough to induce the blank-nut phenotype, due to gene dosage effects. Thus, even in commercial hazel cultivars, a certain proportion of blank-nut formation is common (Beyhan and Marangoz, 2007). Consistent with a cis element in the *SS4* promoter, comparative transcriptome analysis of developing and abortive hazelnut ovules showed that inhibiting gibberellin and activating abscisic acid biosynthesis may contribute to ovule abortion (Cheng et al., 2017). Finally, blank nuts possess heavier shells, implying that photosynthates were preferentially transported to the shell instead of the kernel. In summary, our results suggest that improper cellular localization of SS4 plays a vital role in regulating embryo abortion in hazelnut. These results further verify the biological function of SS4 in seed development through gain or loss mutation. Given that seedlessness is considered a valuable breeding trait in some species, our study provides a new idea for seedless cultivar development.

## Data Availability Statement

All datasets generated for this study are included in the article/**Supplementary Material**.

## Author Contributions

JL and YC contributed to study conception and design, collection and/or assembly of data, data analysis and interpretation, and manuscript writing. SJ, XZ, and HH prepared samples and observed starch granules in cotyledons.

## Funding

This study was supported by grants from the National Natural Science Foundation of China (No. 31670681; 31770723) and the Science and Technology Research Project of The Education Department of Jilin Province (No. JJKH20191012KJ; JJKH20190996KJ).

## Conflict of Interest

The authors declare that the research was conducted in the absence of any commercial or financial relationships that could be construed as a potential conflict of interest.
