# Supplementary figures and images for: Whole-Genome Re-Sequencing of Corylus heterophylla Blank-Nut Mutants Reveals Sequence Variations in Genes Associated With Embryo Abortion
Source: Front Plant Sci. 2019 Nov 13;10:1465. doi: 10.3389/fpls.2019.01465 (PMC6863972; doi:10.3389/fpls.2019.01465)

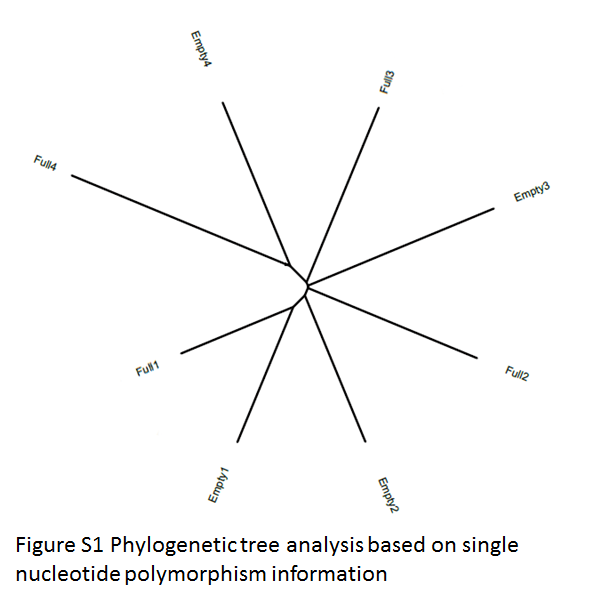

Supplement: Supplementary file 5 [file Image_1.tif]
